# The aberrant dynamic amplitude of low-frequency fluctuations in melancholic major depressive disorder with insomnia

**DOI:** 10.3389/fpsyt.2022.958994

**Published:** 2022-08-22

**Authors:** Zijing Deng, Xiaowei Jiang, Wen Liu, Wenhui Zhao, Linna Jia, Qikun Sun, Yu Xie, Yifang Zhou, Ting Sun, Feng Wu, Lingtao Kong, Yanqing Tang

**Affiliations:** ^1^Brain Function Research Section, The First Affiliated Hospital of China Medical University, Shenyang, China; ^2^Department of Psychiatry, The First Affiliated Hospital of China Medical University, Shenyang, China; ^3^Department of Radiology, The First Affiliated Hospital of China Medical University, Shenyang, China; ^4^Department of Radiation Oncology, The First Affiliated Hospital of China Medical University, Shenyang, China; ^5^Department of Gerontology, The First Affiliated Hospital of China Medical University, Shenyang, China

**Keywords:** melancholic depression, resting-state, magnetic resonance imaging, the dynamic amplitude of low-frequency fluctuation, sleep disturbance, insomnia

## Abstract

**Background:**

Insomnia is considered one of the manifestations of sleep disorders, and its intensity is linked to the treatment effect or suicidal thoughts. Major depressive disorder (MDD) is classified into various subtypes due to heterogeneous symptoms. Melancholic MDD has been considered one of the most common subtypes with special sleep features. However, the brain functional mechanisms in melancholic MDD with insomnia remain unclear.

**Materials and methods:**

Melancholic MDD and healthy controls (HCs, *n* = 46) were recruited for the study. Patients were divided into patients with melancholic MDD with low insomnia (mMDD-LI, *n* = 23) and patients with melancholic MDD with high insomnia (mMDD-HI, *n* = 30), according to the sleep disturbance subscale of the 17-item Hamilton Depression Rating Scale. The dynamic amplitude of low-frequency fluctuation was employed to investigate the alterations of brain activity among the three groups. Then, the correlations between abnormal dALFF values of brain regions and the severity of symptoms were investigated.

**Results:**

Lower dALFF values were found in the mMDD-HI group in the right middle temporal gyrus (MTG)/superior temporal gyrus (STG) than in the mMDD-LI (*p* = 0.014) and HC groups (*p* < 0.001). Melancholic MDD groups showed decreased dALFF values than HC in the right middle occipital gyri (MOG)/superior occipital gyri (SOG), the right cuneus, the bilateral lingual gyrus, and the bilateral calcarine (*p* < 0.05). Lower dALFF values than HC in the left MOG/SOG and the left cuneus in melancholic MDD groups were found, but no significant difference was found between the mMDD-LI group and HC group (*p* = 0.079). Positive correlations between the dALFF values in the right MTG/STG and HAMD-SD scores (the sleep disturbance subscale of the HAMD-17) in the mMDD-HI group (*r* = 0.41, *p* = 0.042) were found. In the pooled melancholic MDD, the dALFF values in the right MOG/SOG and the right cuneus (*r* = 0.338, *p* = 0.019), the left MOG/SOG and the left cuneus (*r* = 0.299, *p* = 0.039), and the bilateral lingual gyrus and the bilateral calcarine (*r* = 0.288, *p* = 0.047) were positively correlated with adjusted HAMD scores.

**Conclusion:**

The occipital cortex may be related to depressive symptoms in melancholic MDD. Importantly, the right MTG/STG may play a critical role in patients with melancholic MDD with more severe insomnia.

## Introduction

Major depressive disorder (MDD) is a widely distributed, heterogeneous, and disabling psychiatric disease (1, 2), characterized by different clinical manifestations, such as persistently depressed mood, anhedonia, low self-esteem, somatization, weight change, cognitive dysfunction, retardation, and sleep disturbances (1). Sleep disturbances, particularly insomnia, are prevalent and prodromal clinical characteristics in MDD (3), and 92% of patients with major depressive episodes reported substantial sleep complaints (4). Insomnia, as one of the main manifestations of sleep disturbances, is not only an important risk factor for developing depression (5, 6) but also for recurrence (7), and its severity is associated with the quality of life (8), the severity of depression (8), the therapeutic effect (9, 10), and suicidal thoughts (11, 12). In addition, the link between MDD and insomnia showed a strong bond based on substantial shared genetic liability (13, 14).

Previous research has shown a significant relationship between sleep disturbance (insomnia especially) and depression (15, 16). Furthermore, several neuroimaging studies have also found objective differences in brain regions and changes in neural activity in patients with MDD (17–21) and insomnia individuals (22–25) by using distinctive imaging approaches, such as the amplitude of low-frequency fluctuation (ALFF), functional connectivity (FC), and regional homogeneity (ReHo). These studies implied that neuroimaging characteristics can correctly reflect individual sleep disturbances and performance and could be used as diagnostic biomarkers of MDD (26). There is also some research into the underlying brain mechanisms of patients with MDD with sleep disturbances using resting-state functional MRI (rs-fMRI). Carried out using various methods of rs-fMRI, such as ALFF or FC in patients with MDD with insomnia, researchers noticed changes in the activity of some brain regions, such as the salience network, the suprachiasmatic nuclei, and the default mode network (27–29). A study using a multicenter dataset found that the combination of gray matter (GM) density and fractional ALFF can accurately predict individual total sleep disturbance scores of the 17-Item Hamilton Depression Rating Scale (HAMD-17) which includes items 4 (sleep initiation disorder), 5 (sleep maintenance disorder), and 6 (early awakening) of HAMD-17 in patients with MDD (26). According to certain findings, patients with MDD with more severe insomnia had smaller cortical surface areas in several frontoparietal cortical areas (30), and the interaction between depression and insomnia was related to reduced GM volume in the right orbitofrontal cortex (31). Besides, abnormal global FC density in the visual system was considered a biomarker in patients with MDD with insomnia (32). As a result, the underlying biological mechanisms of sleep disruption in MDD are being investigated constantly.

However, these studies mentioned above did not go deep into each subtype of MDD, and the heterogeneity of symptoms may bring inconsistent experimental results. In addition, newer imaging methods can also be used in future research.

As a heterogeneous mental disorder, dividing MDD into subcategories based on diagnostic criteria is essential for identifying underlying pathophysiological mechanisms, and this form of individualized diagnosis is required for precise MDD treatment (26). Melancholic MDD has been viewed as the most serious subtype of MDD (33) with specific clinical symptoms, such as persistent anhedonia, psychomotor disturbances, cognitive impairment, early morning awakening, excessive guilt, and anorexia (34). In addition, there are several biological indicators, such as disturbances in sleep architecture, occurring more frequently in melancholia than in other types of depression (35, 36). Studies also found that patients with melancholic MDD had a higher rate of nightmares, middle, and terminal insomnia than patients with non-melancholic MDD (37, 38). Previous research revealed that patients with melancholic MDD displayed evident sleep-electroencephalogram (EEG) changes, which involved low quantities of slow-wave sleep, disrupted sleep, a short rapid eye movement (REM) latency, and a high REM density compared to non-melancholic depression (36, 39–41). However, limited studies use imaging methods to investigate underlying neuroimaging mechanisms of melancholic MDD with insomnia.

Resting-state functional magnetic resonance imaging (rs-fMRI) is a method to measure spontaneous activity in the brain that has been generally utilized to explore functional changes in the human brain (42). There are also several studies about melancholic MDD. These studies found that the melancholic MDD had distinct fractional ALFF values in the right middle temporal gyrus (MTG)/and bilateral superior occipital gyrus (SOG) (43), lower ReHo values in the right SOG/middle occipital gyrus (MOG) (44), different network homogeneity values in the right posterior cingulate cortex (PCC)/precuneus, right angular gyrus, and the right MTG (45), decreased voxel-mirrored homotopic connectivity values in the fusiform gyrus, PCC, and SOG (46). As a promising approach to rs-fMRI, ALFF has been adopted widely because it can reflect the spontaneous neuronal activity of specific regions (47). Simultaneously, ALFF is also considered to be momentarily stationary during a typical rs-MRI session (48). However, the activity of brain areas is inherently dynamic (49). Therefore, to capture the dynamic properties of distinct brain regions’ activity effectively, the dynamic sliding window method such as dALFF has been adopted in several types of research in contrast to the “static” approaches of rs-fMRI. Furthermore, a previous study demonstrated that dALFF changes are related to changes in EEG band power (50). Recently, many psychiatric illnesses have discovered a significant change in dALFF, such as MDD (51), bipolar disorder (52), Schizophrenia (53), primary insomnia (54), and generalized anxiety disorder (55). For an instance, Staner found that temporal dALFF can predict suicidal thoughts in depressed patients, while static ALFF cannot (16). Nevertheless, the dynamics of brain activity in patients with melancholic MDD with insomnia have not yet been quantified.

Therefore, our goal is to investigate the dynamic spontaneous brain activity in patients with melancholic MDD along with insomnia compared to HCs by employing the dALFF. We assume that melancholic MDD with variable degrees of insomnia may show certain particular dynamic brain functional changes and these features are associated with insomnia symptoms of melancholic MDD.

## Materials and methods

### Participants

In the current research, a total of 53 melancholic MDD individuals and 46 healthy controls (HCs) matched for age, gender, and education were recruited from the Shenyang Mental Health Center and The First Affiliated Hospital of China Medical University. Participants ranged in age from 18 to 40. The Medical Research Ethical Committee of the First Affiliated Hospital of China Medical University has approved the study. Before starting the study, all participants were informed of the purpose of the study and signed an informed consent form.

The diagnosis of melancholic MDD was independently performed by two seasoned psychiatrists, according to the Diagnostic and Statistical Manual of Mental Disorders, fourth edition (DSM-IV) criteria. The inclusion criteria for melancholic MDD were: (1) continuously anhedonia; (2) at least three of the symptoms as follows are required: depressive mood, feeling worse in the morning, early morning awakening, remarkable psychomotor disturbances, severe anorexia or weight loss, and excessive or inappropriate guilt. Patients were enrolled in the research while experiencing a depressive episode with a total of HAMD-17 scores ≥ 17 on the day of the MRI scan. These patients were excluded if diagnosed with other Axis I or Axis II disorder.

HCs with HAMD-17 scores ≤ 7 were recruited from the local community and they had no Axis I or Axis II disorder. Meanwhile, their first-degree relatives could not have any family history of psychiatric disorders.

The following exclusion criteria applied to all participants: history of neurological such as head injury, stroke, seizures, or transient ischemic attack; caffeine, drug, or alcohol abuse; and pregnancy.

### Assessments

The depression severity was evaluated by HAMD-17 (56) and the anxiety severity was measured based on the Hamilton Anxiety Rating Scale (HAMA) (57). Insomnia symptoms were evaluated using the insomnia subscale of the HAMD-17 (HAMD-SD) (22, 26, 58), which involved items 4 (sleep initiation disorder), 5 (sleep maintenance disorder), and 6 (early awakening). The scores of the HAMD insomnia subscale and sleep diary data are widely considered to be well correlated, which has been a global measure of insomnia severity in depressive disorders (59). The adjusted HAMD scores meant the HAMD-17 scores after the omission of the sleep subscale scores (27, 32). Patients with melancholic MDD were separated into patients with melancholic MDD with low insomnia group (mMDD-LI, HAMD-SD ≤ 3, *n* = 23) and patients with melancholic MDD with high insomnia (mMDD-HI, HAMD-SD ≥ 4, *n* = 30), according to the HAMD-SD.

### Image acquisition

The rs-MRI functional images were acquired by adopting a 3.0 T GE SIGNA MRI system at the Image Institute of The First Affiliated Hospital of China Medical University, Shenyang, China. The following are the parameters that were obtained using a gradient echo-planar imaging (EPI) sequence: repetition time (TR) = 2,000 ms, echo time (TE) = 40 ms, field of view (FOV) = 240 × 240 mm^2^, flip angle (FA) = 90°, image matrix size = 64 × 64, slices = 35, slice thickness = 3 mm, spacing between slices = 3 mm. Participants were required to wear earplugs and use foam pads to reduce scanner noise and head motion, and their head was fixedly positioned during scan time. The resting state meant that participants were not doing any cognitive tasks during the scan time and they were asked to keep relaxed and awake with their eyes closed while simultaneously keeping their minds blank. After completing scanning, we obtained a total of 200 volumes of images.

### Image pre-processing

Images were processed by applying the Statistical Parametric Mapping 12 (SPM12)^1^ and the Data Processing Assistant for Resting-State fMRI (DPABI 4.1, Advanced edition) (60) based on the custom code written in MATLAB. To maintain the stability of the initial signal, the first 10 volumes of each participant’s scanned data were eliminated. Then, the remaining 190 images were adjusted for slice-timing and head motion. Participants with the translation of more than 2 mm or rotation of more than 2° of head motion in each direction were not included in the present study. The mean framewise displacement (FD) was used to measure the scrubbing-related micro-head motion of each participant. Additionally, no statistical difference existed in mean FD when comparing the three groups. After realignment, the imaging data corrected were spatially normalized into a standard EPI template in the Montreal Neurological Institute (MNI) space and resampled to a voxel size of 3 mm × 3 mm × 3 mm. The spatial smoothing of the EPI images adopted a 4 mm full width at half maximum (FWHM) Gaussian kernel. The BOLD signals were then detrended to correct a linear trend. Finally, the linear regression of the nuisance covariates was performed to remove the effects, including head motion parameters, cerebrospinal fluid signal, and white matter signal. Normalization of T1 images was also conducted, and details and results are shown in Supplementary Table 3 and Supplementary Figure 3, and pre-processing part.

### Dynamic amplitude of low-frequency fluctuation acquisition

The dynamic ALFF was computed using temporal dynamic analysis (TDA) toolkits in DPABI 4.1 (60) based on a sliding window analysis. The window length which was considered a crucial parameter of resting-state dynamic computation should be larger than 1/*f*_*min*_. The *f*_*min*_ referred to the minimum frequency of the time series. As a shorter window length is more likely to introduce misleading fluctuations in the observed dALFF, a longer window length may fail to discover the potential dALFF. Therefore, a window length of 50 TRs (100 s) was selected to compute the temporal variability of ALFF according to prior research (51, 61). We calculated the ALFF of each subject with a sliding window and obtained the ALFF values of each given voxel, the time series of which were then transformed into a frequency domain with a fast Fourier transform. The square root of the power spectrum of each voxel was computed and summed from 0.01 to 0.08 Hz. The dALFF values were obtained through computing the standard deviation (SD) of ALFF values at each voxel across the sliding window dynamics. SD is a useful and quantified indicator that is generally applied to describe the degree of change in dALFF in the research of temporal dynamic brain activity. Therefore, we used SD as dALFF for the next analysis.

### Statistical analysis

The demographic features and clinical symptoms were analyzed by several statistical methods, such as one-way analysis of variance (ANOVA), two-sample *t*-test, and chi-square test, and *p* < 0.05 was set as significance. The specific use of these statistical methods is shown in Table 1. The relationships between the total HAMD-17 scores, adjusted HAMD scores, HAMD-SD scores, and HAMA scores were explored by Pearson’s correlation in the pooled melancholic MDD groups, and the statistical significance was also set at *p* < 0.05. Images data were analyzed by two software, and two types of correction are used. Altered dynamic ALFF values across the three groups were investigated by ANOVA in DPABI 4.1 and the results were corrected with the Gaussian random field (GRF) one-tailed (62), the threshold of voxel-wise was *p* < 0.001, and the cluster-level was *p* < 0.05. The extracted dALFF values were evaluated by SPSS (Statistical Product and Service Solutions) and the one-way analysis of covariance (ANCOVA) was used to compare differences among three groups when age, gender, years of education, and FD were covariates. Significance was set at *p* < 0.05 after correcting by Bonferroni correction. The partial correlation analysis was conducted to examine the relationship between the altered dALFF values and clinical features (HAMD-SD scores, HAMA scores, and adjusted HAMD scores) in melancholic MDD groups when age, gender, medication, HAMA scores, adjusted HAMD scores, or HAMD-SD scores were used as covariates. Significance was set at *p* < 0.05 after correcting by Bonferroni correction.

**TABLE 1:** Demographic and clinical measurement among three groups.

Measurement	mMDD-LI (*n* = 23)	mMDD-HI (*n* = 30)	HC (*n* = 46)	Statistical value	*P-*value
Age (years)	24.65 ± 5.57	26.83 ± 6.81	25.65 ± 6.08	0.832	0.442*
Gender (male/female)	8/15	6/24	20/26	4.441	0.109^#^
Education (years)	14.70 ± 2.42	14.73 ± 2.27	14.63 ± 1.88	0.022	0.978*
Mean FD	0.11 ± 0.54	0.93 ± 0.39	0.12 ± 0.51	2.061	0.133*
Illness duration (months)	17.39 ± 19.61	13.15 ± 18.28	NA	0.81	0.420
Number of episodes	1.13 ± 0.34	1.17 ± 0.38	NA	−0.36	0.720
HAMD-SD	2.52 ± 0.67	4.87 ± 0.82	NA	−11.18	<0.001
Adjusted HAMD	19.43 ± 2.83	20.20 ± 5.25	NA	−0.68	0.500
HAMD-17	21.96 ± 3.07	25.07 ± 5.45	NA	−2.63	0.012
HAMA	19.61 ± 7.05	24.17 ± 8.24	NA	−2.12	0.039
**Medication**	19 (83%)	19 (63%)	NA	0.171	0.879^#^
Antidepressants	19 (83%)	15 (60%)	NA	NA	NA
Benzodiazepines	13 (57%)	12 (40%)	NA	NA	NA
Medication-free	4 (17%)	11 (37%)	NA	NA	NA

Data are presented as either percentages (%) or means (standard deviations).

^#^P-value for chi-square test, *P-value for one-way ANOVA, the rest P-value for two-sample t-tests. NA stands for not available; mMDD-LI, patients with melancholic MDD with low insomnia group; mMDD-HI, patients with melancholic MDD with high insomnia; HC, healthy control; FD, framewise displacement; HAMD-17, 17-item Hamilton Depression Rating Scale; HAMD-SD, the sleep disturbance factor of the HAMD-17 subscale; adjusted HAMD, HAMD-17 scores after the omission of the sleep subscale scores; HAMA, Hamilton Anxiety Rating Scale.

### Validation analysis

To verify the results of dALFF variability obtained from a sliding window length of 50 TR, we adopted using two other window lengths (30 and 70 TR).

## Results

### Demographic characteristics and clinical symptoms

As Table 1 illustrates age, sex, years of education, and mean FD were not significantly different among the mMDD-LI, mMDD-HI, and HC groups (*p* > 0.05). And there were no significant differences in medication, duration of disease, or number of episodes between the two melancholic MDD groups (*p* > 0.05). Compared with the mMDD-LI, mMDD-HI showed higher HAMD (*p* = 0.012) and HAMA scores (*p* = 0.039). However, the adjusted HAMD scores were not statistically different between the two melancholic MDD groups (*p* = 0.50). In the pooled melancholic MDD, the HAMD-SD scores were significantly positively associated with the HAMD scores (*r* = 0.452, *p* = 0.001) and HAMA scores (*r* = 0.377, *p* = 0.005), but not with the adjusted HAMD scores (*r* = 0.178, *p* = 0.203). The HAMA scores were also positively associated with the HAMD scores (*r* = 0.68, *p* < 0.001) and the adjusted HAMD scores (*r* = 0.629, *p* < 0.001) in the pooled melancholic MDD.

### Differences in dynamic amplitude of low-frequency fluctuation values among three groups

**FIGURE 1:**
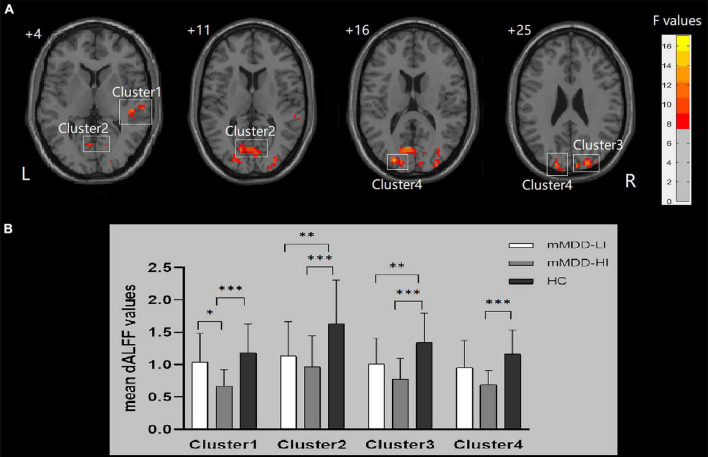
Differences in dALFF values and *post hoc* analysis of dALFF values among three groups. Cluster 1: right MTG/STG, Cluster 2: bilateral lingual gyrus/calcarine; Cluster 3: right middle occipital gyrus/superior occipital gyrus/cuneus; Cluster 4: left middle occipital gyrus/superior occipital gyrus/cuneus. **(A)** Significant difference in dALFF values among the three groups. Significant at *p* < 0.001 after GRF correction. R, right side; L, left side. **(B)** *Post hoc* analysis of dALFF values with significant variations across the three groups. ***p < 0.001 level, **p < 0.01 level, *p < 0.05 level, Bonferroni correction.

Significant group differences of dALFF were found in the right MTG/superior temporal gyrus (STG), the bilateral MOG/SOG, the bilateral cuneus, the bilateral calcarine, and the bilateral lingual gyrus (*p* < 0.001, GRF correction). The ANCOVA revealed that the mMDD-HI group displayed lower dALFF values in the right MTG/STG than the mMDD-LI group (*p* = 0.014, Bonferroni correction) and HC group (*p* < 0.001, Bonferroni correction). Meanwhile, two melancholic MDD groups showed lower dALFF values than HC in the right MOG/SOG, the right cuneus, the bilateral lingual gyrus, and the bilateral calcarine (*p* < 0.05, Bonferroni correction). The two melancholic MDD groups had lower dALFF values than HC in the left MOG/SOG and the left cuneus, although the difference between the mMDD-LI group and HC group was not statistically significant (*p* = 0.079, Bonferroni correction). Table 2 and Figure 1 illustrate these findings.

**TABLE 2:** Brain regions showing significant group differences in dALFF values.

Cluster	Hemisphere	Brain regions	Cluster size (voxels)	MINI coordinates (x, y, z)			*F*-values
1	Right	Middle temporal gyrus/superior temporal gyrus	43	48	−27	3	12.913
2	Bilateral	Calcarine/lingual gyrus	92	−6	−72	15	11.936
3	Right	Middle occipital gyrus/superior occipital gyrus/cuneus	81	21	−96	9	14.964
4	Left	Middle occipital gyrus/superior occipital gyrus/cuneus	135	−24	−87	15	12.318

MINI, Montreal Neurological Institute; dALFF, the dynamic amplitude of low-frequency fluctuation.

### Correlations between dynamic amplitude of low-frequency fluctuation values and clinical characteristics

The partial correlation analysis revealed that the dALFF values in the right MTG/STG were positively correlated with HAMD-SD scores in the mMDD-HI group when age, gender, medication, and adjusted HAMD scores, and HAMA scores were covariates (*r* = 0.41, *p* = 0.042). In the pooled melancholic MDD, the dALFF values in the right MOG/SOG and the right cuneus were significantly positively correlated with adjusted HAMD scores (*r* = 0.338, *p* = 0.019), the dALFF values in the left MOG/SOG and the left cuneus were positively correlated with adjusted HAMD scores (*r* = 0.299, *p* = 0.039), and the dALFF values in the bilateral lingual gyrus and the bilateral calcarine were positively correlated with adjusted HAMD scores (*r* = 0.288, *p* = 0.047) when age, gender, medication, HAMD-SD scores, HAMA scores were as covariates. The above results are shown in Tables 3, 4. No other correlation was found.

**TABLE 3:** The partial correlation analysis between the dALFF values and adjusted HAMD scores in the pooled melancholic MDD.

Cluster	Hemisphere	Brain regions	Cluster size (voxels)	MINI coordinates (x, y, z)			*r*	*p*
2	Bilateral	Calcarine/lingual gyri	92	−6	−72	15	0.288	0.047
3	Right	Middle occipital gyrus/superior occipital gyrus/cuneus	81	21	−96	9	0.338	0.019
4	Left	Middle occipital gyrus/superior occipital gyrus/cuneus	135	−24	−87	15	0.299	0.039

Covariates included age, gender, medication, HAMD-SD scores, and HAMA scores. MINI, Montreal Neurological Institute; dALFF, the dynamic amplitude of low-frequency fluctuation.

**TABLE 4:** The partial correlation analysis between the dALFF values and HAMD-SD scores in patients with melancholic MDD with high insomnia.

Cluster	Hemisphere	Brain regions	Cluster size (voxels)	MINI coordinates (x, y, z)			*r*	*p*
1	Right	Middle temporal gyrus/superior temporal gyrus	43	48	−27	3	0.41	0.042

Covariates included age, gender, medication, adjusted HAMD scores, and HAMA scores. MINI, Montreal Neurological Institute; dALFF, the dynamic amplitude of low-frequency fluctuation; HAMD-SD, the sleep disturbance factor of the HAMD-17 subscale.

### Validation results

In the present study, we validated our results by using different sliding window lengths (30 and 70 TR). Finally, the relevant results were presented in Supplementary Tables 1, 2 and Supplementary Figures 1, 2.

## Discussion

In the present study, we investigated the neuroimaging features of melancholic MDD with varying degrees of insomnia by adopting the dynamic sliding window method. Our research found that the mMDD-HI group displayed significantly decreased dALFF values in the right MTG/STG than the mMDD-LI and HC groups. Furthermore, the dALFF values in the right MTG/STG were significantly positively correlated with HAMD-SD scores in the mMDD-HI group. We also found two melancholic MDD groups showed decreased dALFF values than HC in the right MOG/SOG, the right cuneus, the bilateral lingual gyrus, and the bilateral calcarine. The two melancholic MDD groups had lower dALFF values than HC in the left MOG/SOG and the left cuneus, but the difference between the mMDD-LI group and HC group was not statistically significant. Meanwhile, the dALFF values in the bilateral MOG/SOG, the bilateral cuneus, the bilateral lingual gyrus, and the bilateral calcarine were significantly positively correlated with adjusted HAMD scores in the pooled melancholic MDD. These findings suggested that the bilateral occipital cortex may be associated with the neuroimaging mechanisms of melancholic MDD depressive symptoms other than insomnia, while the temporal cortex may play an important role in more severe insomnia symptoms in patients with melancholic MDD.

In the present study, the mMDD-HI group revealed alterations of dALFF in the right temporal cortex. Meanwhile, the right temporal cortex may be associated with more severe insomnia symptoms in patients with melancholic MDD. Previous studies also speculated that functional alterations in the right MTG/STG may be a neurobiological characteristic of melancholic MDD (44, 45). Some studies found GM hypertrophies (63) and diminished ALFF in the right MTG (64) in patients with insomnia disorder. The altered fALFF brain areas of patients with insomnia were found in the right STG, which was unaffected by age and gender factors (65). This evidence suggests that the right MTG/STG may play an important role in insomnia symptoms of melancholic depression. Previous studies have proposed the hyperarousal hypothesis to explain the mechanism of insomnia, which is the result of hyperarousal in emotional or cognitive networks (66, 67). Furthermore, emotional and cognitive functions, such as emotional regulation, social cognition, and the process of memory, have been considered to be associated with the temporal lobe (55, 68), which may explain the hyperarousal hypothesis. Hence, we speculated that the abnormal dALFF values in the right temporal cortex may be one of the neuroimaging mechanisms of high insomnia symptoms in melancholic MDD.

However, there are also studies that are inconsistent with our results. They found several brain regions, such as the left inferior temporal gyrus (32), the posterior parahippocampal/hippocampal gyrus (32), frontoparietal cortical areas (30), caudate nuclei (69), and the anterior insular subregions (70), were associated with insomnia symptoms in patients with MDD. We went on to think about the reasons for these differences, which may be caused by distinct rs-fMRI methods or the heterogeneous symptoms of patients with MDD. This suggests that we need to expand the sample size, pay more attention to the heterogeneity of MDD, and adopt newer methods for analysis in future.

In the current study, we also discovered that melancholic MDD caused changes in dALFF values in the occipital cortex. Furthermore, these findings revealed that the occipital cortex may be associated with depressive characteristics. In recent years, multiple studies reported that patients with melancholic MDD manifested considerably lower values in the MOG/SOG than patients with non-melancholic MDD or HCs by applying rs-fMRI neuroimaging methods (44–46). However, there is not much literature on melancholic MDD, and we found similar results in other studies on MDD. Another research also found lower ALFF in the bilateral occipital cortex in treatment-free MDD subjects (71). The results indicate that decreased dALFF values of the calcarine in MDD are essentially consistent with findings from another study, where lower ALFF values were investigated in the left calcarine (27). Apart from functional changes, melancholic depression involves structural abnormalities, according to growing neuroimaging research evidence. Previous studies have found that patients with melancholic MDD showed asymmetrical cerebrospinal fluid (CSF) volume enlargement in the Sylvian fissure region (72, 73). The increased CSF volume may cause occipital bending, which may result in functional changes in the occipital cortex (74). These present findings are in line with the results of the previous studies. Therefore, these results revealed that alterations in the occipital cortex may be specific in melancholic MDD.

Besides, correlation analysis results show that the occipital cortex may be associated with depressive traits. The lingual gyrus and cuneus, located in the medial occipital lobe, are necessary for both basic and advanced visual processing (75). The function of the occipital cortex is to process initial visual stimuli, consolidate information into visual working memory (76), and revolve in the consciousness of facial expressions (77), and its aberrant changes may explain working memory impairment in melancholic MDD (78). A study demonstrated functional changes in the occipital cortex when depressed adolescents are presented with sad faces, and the occipital cortex is important to the interaction of depression status, attention, and mood processing (79). In addition, the functional stability in the middle occipital gyri (MOG) was negatively linked with depressive symptoms, which could predict long-term recovery in depressive symptoms in patients with MDD (80). As a result, we hypothesized that the occipital cortex maybe plays a major role in the neuroimaging mechanisms of other depressive symptoms more than insomnia symptoms in melancholic.

In addition, we also observed that the mMDD-HI group showed lower dALFF values in all brain regions found in the current study compared to the mMDD-LI group and HC group, although certain differences did not achieve statistical significance. However, previous similar investigations using different imaging methods have yielded inconsistent results (27, 32). As a result, future studies will require a larger sample size to verify these results.

From our research, we observed several limitations. First, HAMD was adopted to evaluate the severity of insomnia, which may cause an incomplete assessment. In further studies, we could apply more objective and subjective measurements, such as Pittsburgh Insomnia Rating Scale or polysomnography, to improve the precision of the severity of insomnia assessments. Second, most patients had been on medicine before the scan and the drug-related effects cannot be excluded. In subsequent statistical analysis, we attempted to limit the impact of medication on the results by taking medication as a covariance. More medication-free patients should be enrolled in our research. Finally, our study was a cross-sectional study with a small sample size for each subgroup, and the sample size should be expanded in future, and relevant longitudinal work should be conducted.

In conclusion, the present study investigated brain functional alterations in melancholic MDD with insomnia. We found that the occipital cortex may be related to depressive symptoms in those with melancholic MDD, which further supports the findings of previous imaging studies on melancholic MDD. Moreover, the major finding of the present study revealed that the right temporal cortex was associated with more severe insomnia symptoms in melancholic MDD, which is consistent with our earlier hypothesis, and it suggested that the right temporal cortex could be a biomarker for melancholic MDD with high insomnia.

## Data availability statement

The raw data supporting the conclusions of this article will be made available by the corresponding author.

## Ethics statement

The studies involving human participants were reviewed and approved by the Medical Research Ethical Committee of The First Affiliated Hospital of China Medical University. The patients/participants provided their written informed consent to participate in this study. Written informed consent was obtained from the individual(s) for the publication of any potentially identifiable images or data included in this article.

## Author contributions

ZD performed investigation, data processing, statistical analyses, and visualization, and wrote the original draft. XJ was responsible for conceptualization, data curation, writing of original draft, and funding acquisition. WL wrote the original draft. WZ and LJ performed investigation and data processing. QS acquired the data. YX was responsible for statistical analyses. YZ and TS validated the results. FW and LK recruited patients, confirmed the diagnosis, and acquired funding. YT was responsible for conceptualization, project administration, and funding acquisition. All authors reviewed and approved the manuscript.

## Footnotes

1
www.fil.ion.ucl.ac.uk/spm/software/spm12
