# Supplementary material for: The aberrant dynamic amplitude of low-frequency fluctuations in melancholic major depressive disorder with insomnia
Source: Front Psychiatry. 2022 Aug 22;13:958994. doi: 10.3389/fpsyt.2022.958994 (PMC9441487; doi:10.3389/fpsyt.2022.958994)
Supplement: Supplementary file 1 [file Data_Sheet_1.docx]

**Supplementary materials for**

**“The Aberrant Dynamic Amplitude of Low-Frequency Fluctuations in Melancholic Major Depressive Disorder with Insomnia”**

**Zijing Deng^1,2†^, Xiaowei** **Jiang^1,3†^, Wen Liu^1,2^, Wenhui Zhao^1,2^, Linna Jia^1,2^, Qikun Sun^4^, Yu Xie****^1^, Yifang Zhou^1,2^, Ting Sun^1,2^, Feng Wu****^1,2^, Lingtao Kong^1,2^, Yanqing Tang^2,5*^**

^1^Brain Function Research Section, The First Affiliated Hospital of China Medical University, Shenyang, Liaoning, China.

^2^Department of Psychiatry, The First Affiliated Hospital of China Medical University Shenyang, Liaoning, China.

^3^Department of Radiology, The First Affiliated Hospital of China Medical University Shenyang, Liaoning, China.

^4^Department of Radiation Oncology, The First Affiliated Hospital of China Medical University Shenyang, Liaoning, China.

^5^Department of Gerontology, The First Affiliated Hospital of China Medical University Shenyang, Liaoning, China.

**^†^These authors have contributed equally to this work and share the first authorship**

*** Correspondence:**

**Yanqing Tang**

**tangyanqing@cmu.edu.cn**

**Keywords: melancholic depression, resting-state, magnetic resonance imaging, the dynamic amplitude of low-frequency fluctuation, sleep disturbance, insomnia.**

| Cluster | Hemisphere | Brain regions | Cluster size  （voxels） | MINI coordinates  (x, y, z) | | | F values |
| --- | --- | --- | --- | --- | --- | --- | --- |
| 1 | Left | Middle temporal gyrus / superior temporal gyrus | 124 | -45 | 0 | -15 | 17.283 |
| 2 | Left | Lingual gyrus / calcarine / middle occipital gyrus / superior occipital gyrus / cuneus | 292 | -24 | -87 | 15 | 12.799 |
| 3 | Right | Middle temporal gyrus / superior temporal gyrus | 150 | 63 | -24 | 3 | 18.442 |
| 4 | Right | Middle occipital gyrus / superior occipital gyrus / cuneus | 138 | 21 | -93 | 9 | 14.036 |

**Table S1** Brain regions showing significant group differences in dALFF variability (30 TRs)

Note: MINI, Montreal Neurological Institute; dALFF, the dynamic amplitude of low-frequency fluctuation.

| Cluster | Hemisphere | Brain regions | Cluster size  （voxels） | MINI coordinates  (x, y, z) | | | F values |
| --- | --- | --- | --- | --- | --- | --- | --- |
| 1 | Bilateral | Calcarine | 69 | -3 | -72 | 15 | 11.525 |
| 2 | Right | Middle occipital gyrus / superior occipital gyrus / cuneus | 108 | 33 | -87 | 21 | 15.131 |
| 3 | Left | Middle occipital gyrus / superior occipital gyrus / cuneus | 55 | -18 | -87 | 36 | 14.309 |
| 4 | Right | Paracentral lobule | 44 | 6 | -27 | 69 | 11.210 |

**Table S2** Brain regions showing significant group differences in dALFF variability (70 TRs)

Note: MINI, Montreal Neurological Institute; dALFF, the dynamic amplitude of low-frequency fluctuation.

**Table S3** Brain regions showing significant group differences in dALFF among the three groups (normalization by DARTEL)

| Cluster | Brain regions | Cluster size  （voxels） | | MINI coordinates  (x, y, z) | | | | F values |
| --- | --- | --- | --- | --- | --- | --- | --- | --- |
| 1 | Right lingual gyrus / right fusiform gyrus | | 71 | | 15 | -78 | -9 | 18.031 |
| 2 | Bilateral calcarine / right middle occipital gyrus / right superior occipital gyrus / bilateral cuneus / left lingual gyrus | | 227 | | 24 | -90 | 24 | 14.582 |
| 3 | Right postcentral gyrus | | 51 | | 66 | -9 | 18 | 18.131 |
| 4 | Left middle occipital gyrus / left superior occipital gyrus | | 35 | | -15 | -87 | 24 | 13.676 |
| 5 | Right supplementary motor area / bilateral paracentral lobule | | 66 | | 9 | -42 | 51 | 13.513 |

Note: MINI, Montreal Neurological Institute; dALFF, the dynamic amplitude of low-frequency fluctuation.

Sample size: healthy controls, n = 36; mMDD-LI, n=20; mMDD-HI, n=29.

**Figure S1** Post hoc analysis of dALFF variability (30 TRs) with significant variations across the three groups.

****p* < 0.001 level, ***p* < 0.01 level, **p* < 0.05 level, Bonferroni correction.

Cluster 1: left middle temporal gyrus / superior temporal gyrus, Cluster 2: left lingual gyrus / calcarine / middle occipital gyrus / superior occipital gyrus / cuneus; Cluster 3: right middle temporal gyrus / superior temporal gyrus; Cluster 4: right middle occipital gyrus / superior occipital gyrus / cuneus.

**Figure S2** Post hoc analysis of dALFF variability (70 TRs) with significant variations across the three groups.

****p* < 0.001 level, ***p* < 0.01 level, **p* < 0.05 level, Bonferroni correction.

Cluster 1: bilateral calcarine; Cluster 2: right middle occipital gyrus / superior occipital gyrus / cuneus; Cluster 3: left middle occipital gyrus / superior occipital gyrus / cuneus; Cluster 4: right paracentral lobule.

**Figure S3** Post hoc analysis of dALFF (50 TRs, normalized by the DARTEL) among the three groups

****p* < 0.001 level, ***p* < 0.01 level, **p* < 0.05 level, Bonferroni correction.

Cluster 1: right lingual gyrus / right fusiform gyrus; Cluster 2: bilateral calcarine / right middle occipital gyrus / right superior occipital gyrus / bilateral cuneus / left lingual gyrus; Cluster 3: right postcentral gyrus; Cluster 4: left middle occipital gyrus / left superior occipital gyrus; Cluster 5: right supplementary motor area / bilateral paracentral lobule.

Sample size: healthy controls, n = 36; mMDD-LI, n=20; mMDD-HI, n=29.

Pre-Processing

Images were processed by applying the Statistical Parametric Mapping 12 (SPM12, www.fil.ion.ucl.ac.uk/spm/software/spm12) and the Data Processing Assistant for Resting-State fMRI (DPABI 4.1, Advanced edition) based on the custom code written in MATLAB. To maintain the stability of the initial signal, the first 10 volumes of each participant's scanned data were eliminated. Then, the remaining 190 images were adjusted for slice-timing and head-motion. Participants with the translation of more than 2 mm or rotation of more than 2° of head motion in each direction were not included in the present study. The mean framewise displacement (FD) was used to measure the scrubbing-related micro-head motion of each participant. After realignment, resting state images were normalized to T1 images using DARTEL algorithm for resampling (3*3*3) mm and transformed to MNI template. Then we used 4 mm full width and half height Gaussian kernel for smoothing. The BOLD signals were then detrended to correct a linear trend. Finally, the linear regression of the nuisance covariates was performed to remove the effects, including head motion parameters, cerebrospinal fluid signal, and white matter signal.
