# Supplementary figures and images for: The aberrant dynamic amplitude of low-frequency fluctuations in melancholic major depressive disorder with insomnia
Source: Front Psychiatry. 2022 Aug 22;13:958994. doi: 10.3389/fpsyt.2022.958994 (PMC9441487; doi:10.3389/fpsyt.2022.958994)

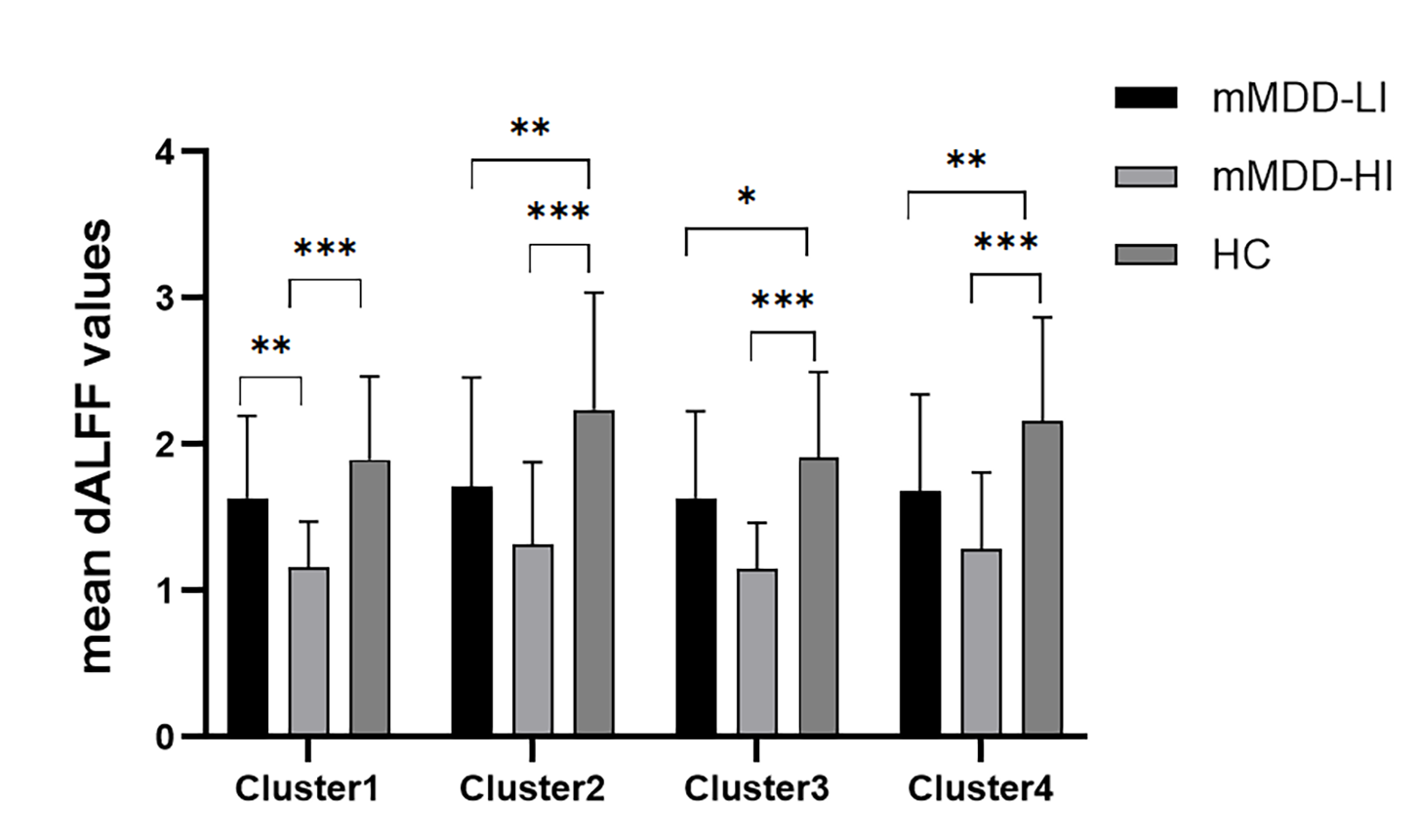

Supplement: Supplementary file 2 [file Image_1.TIF]

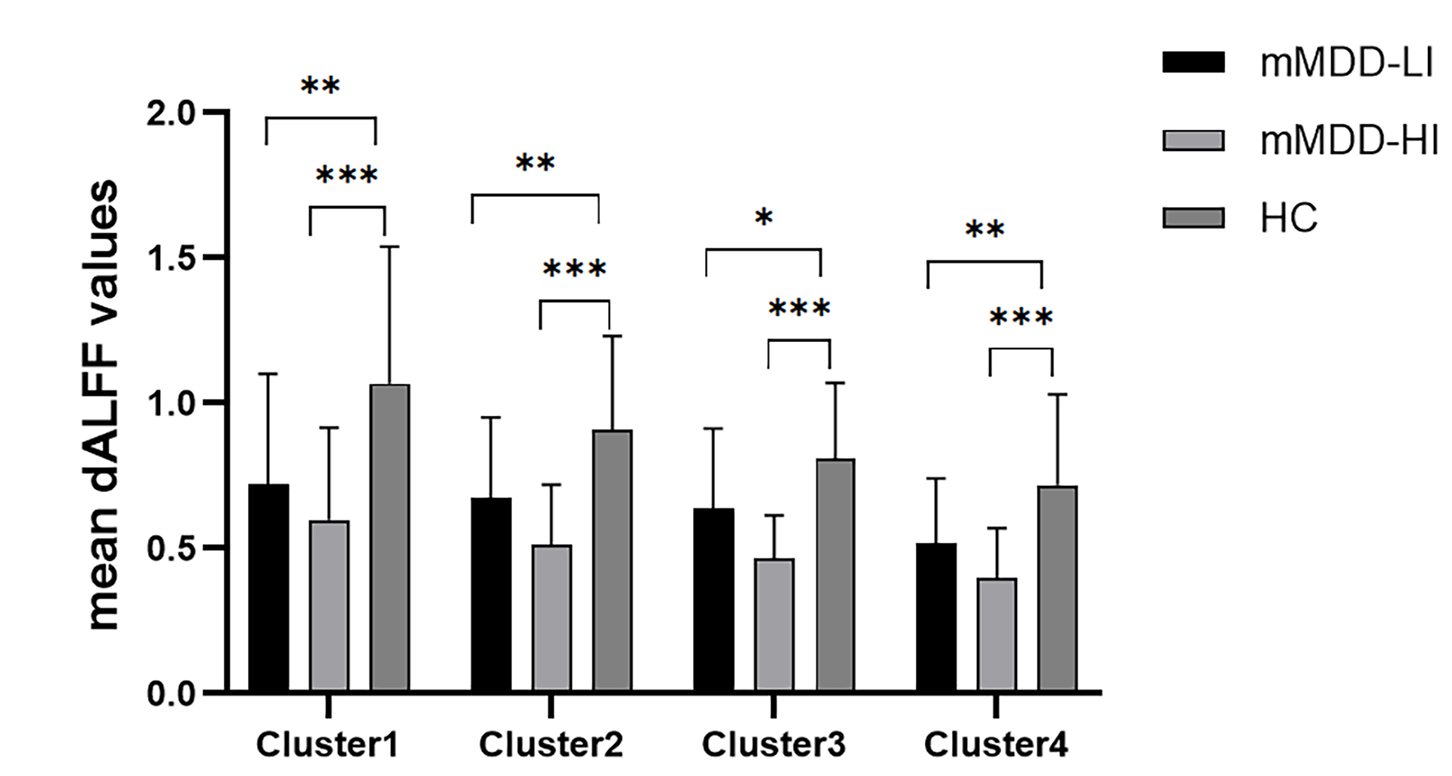

Supplement: Supplementary file 3 [file Image_2.TIF]

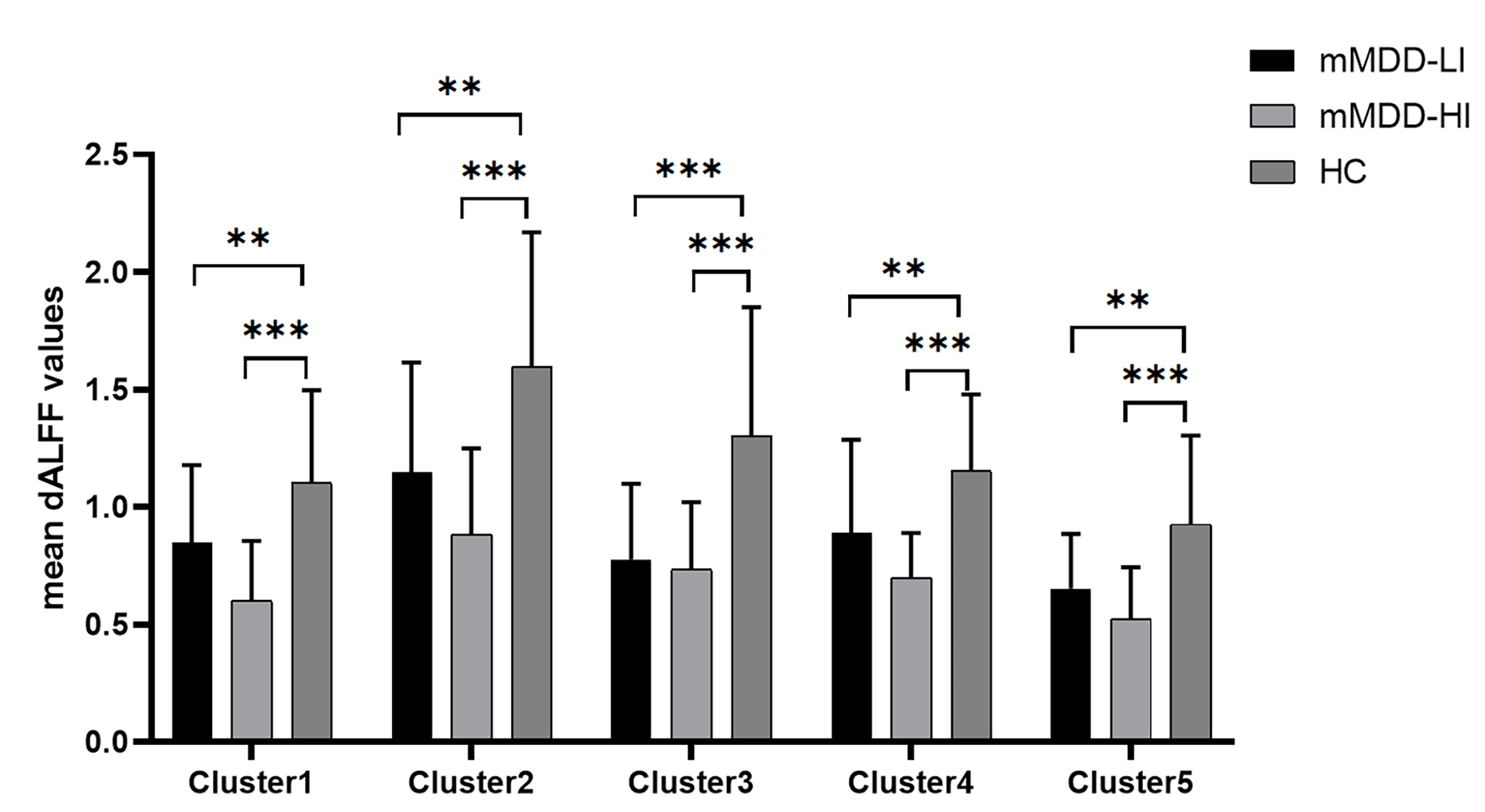

Supplement: Supplementary file 4 [file Image_3.TIF]
